# Two new species of *Pselaphodes* Westwood and new record of *Taiwanophodes minor* Hlaváč from South China (Coleoptera, Staphylinidae, Pselaphinae)

**DOI:** 10.3897/zookeys.175.2664

**Published:** 2012-03-16

**Authors:** Zi-Wei Yin, Li-Zhen Li, Mei-Jun Zhao

**Affiliations:** 1 Department of Biology, College of Life and Environmental Sciences, Shanghai Normal University, Shanghai, 200234, P. R. China

**Keywords:** Staphylinidae, Pselaphinae, *Pselaphodes*, *Taiwanophodes*, taxonomy, new species, new record, South China

## Abstract

Two new species, *Pselaphodes linae* Yin & Li, **sp. n.** (Hainan, Fujian) and *Pselaphodes shii* Yin & Li, **sp. n.** (Hainan) are described from South China. *Taiwanophodes minor* Hlaváč is reported from outside Taiwan for the first time. Illustrations of major diagnostic features are provided for all treated taxa. The latest key to Chinese *Pselaphodes* is modified to include the new species.

## Introduction

A number of tyrine specimens have been submitted for determination since the publication of our previous papers of the genus *Pselaphodes* Westwood (Yin et al. 2010, 2011, 2012). Among this material were two new *Pselaphodes* species, and two males and three females of *Taiwanophodes minor* Hlaváč, a species previously known only from Taiwan. This information is reported herein.

## Methods

The terminology of foveal system follows Chandler (2001), except for using ‘ventrite’ instead of ‘sternite’ when concerning meso- and metathoracic structures.

A slash (/) is used to separate lines on the same label, and a double slash (//) is used to separate different labels on the same pin.

Measurements are in millimeter, the following acronyms are used in the text: **AL**–length of the abdomen along the midline; **AW**–maximum width of the abdomen; **BL**–length of the body (= HL + PL + EL + AL); **EL**–length of the elytra along the sutural line; **EW**–maximum width of the elytra; **HL**–length of the head from the anterior clypeal margin to the occipital constriction; **HW**–width of the head across eyes; **PL**–length of the pronotum along the midline; **PW**–maximum width of the pronotum.

The type series are deposited in the Insect Collection of Shanghai Normal University, Shanghai, China (**SNUC**).

## Taxonomy

### Pselaphodes linae

Yin & Li sp. n.

urn:lsid:zoobank.org:act:7C845FB3-6BEA-4CEC-91CC-75805DF662BE

http://species-id.net/wiki/Pselaphodes_linae

Figs 1 2

#### Type locality.

 China, Hainan Province, Wuzhishan Mountain.

#### Type material

(11 ♂♂, 5 ♀♀)**.** HOLOTYPE: ♂, labeled ‘**CHINA:** Hainan Prov. / Wuzhishan Mt. / Guzhandao / ca. 640 m, 30.xi.2009 / 18.86657°N, 109.68285°E / Mei-Ying Lin leg. // [red] HOLOTYPE / *Pselaphodes linae* Yin & Li / det. 2012, SNUC’. PARATYPES (all bear the following label: ‘[yellow] PARATYPE / *Pselaphodes linae* Yin & Li / det. 2012, SNUC’): 2 ♂♂, 3 ♀♀, same label data as holotype; 2 ♂♂, 1 ♀, labeled ‘**CHINA:** Hainan Prov. / Yinggeling N. R. / Daoyin–Shenfu / ca. 425 m, 22.xi.2009 / Mei-Ying Lin leg.’; 1 ♀, same label data, except ‘14.IV.2010 / 18.98399°N, 109.33585°E’; 5 ♂♂, labeled ‘**CHINA:** Hainan Prov. / Baisha, Yuanmen / Yinggeling N. R. / Yinggezui / 660 m, 26.iv.2011 / Wen-Xuan Bi leg.’; 1 ♂, labeled ‘**CHINA:** Fujian Prov. / Nanjing County / Huboliao / ca. 200 m, 22.xi.2008 / Gan-Yan Yang leg. (beating)’.

#### Diagnosis.

 Reddish brown; medium-sized; genae rounded; antennomeres IX–XI strongly modified; pronotum with rounded lateral margins; with thin and long metaventral processes (in lateral view); pro- and metatibiae sinuate; aedeagus with asymmetric median lobe and small parameres.

#### Description.

**Figure 1.**
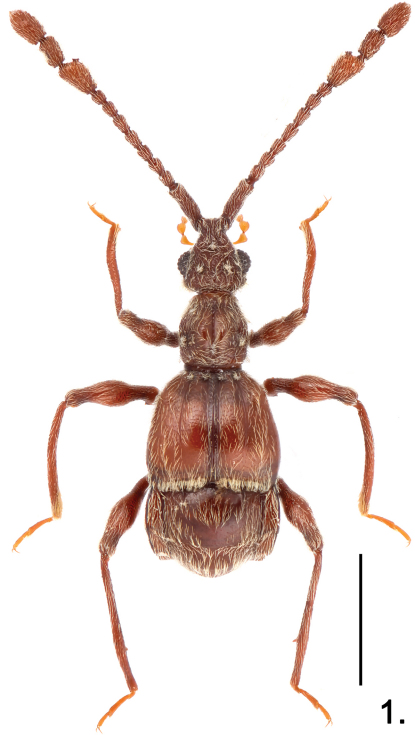
Habitus of *Pselaphodes linae*. Scale: 1.0 mm.

**Figure 2.**
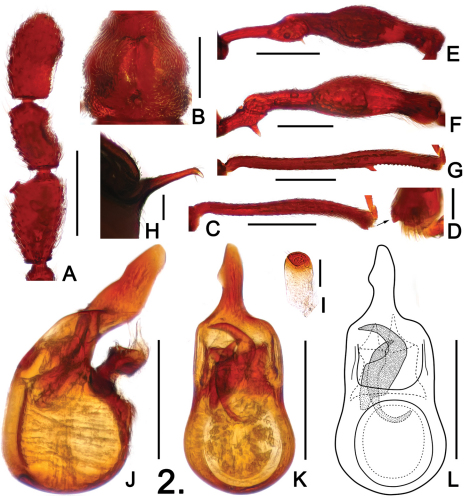
Details of male *Pselaphodes linae*. **A** right antennal club **B** pronotum **C** protibia **D** apex of protibia, enlarged **E** mesotrochanter and mesofemur **F** metatrochanter and metafemur **G** metatibia **H** metaventral process, in lateral view **I** sternite IX **J** aedeagus, in lateral view **K–L** same, in dorsal view. Scales: A, B, C, E, F, G = 0.3 mm, H, I = 0.1 mm, D = 0.05 mm, J, K, L = 0.2 mm.

 Male (Fig. 1). Length 2.65–2.87. Head as long as wide, HL 0.56–0.58, HW 0.56–0.58; eyes large, each composed of about 40 facets. Antennal clubs as in Fig. 2A. Pronotum (Fig. 2B) slightly longer than wide, PL 0.55–0.56, PW 0.50–0.52, with round lateral margins. Elytra wider than long, EL 0.80–0.81, EW 0.99–1.04. Metaventral horn-like processes long (Fig. 2H). Legs having protibiae with blunt and slightly serrate apical projection (Figs 2C, D); mesotrochanters with minute ventral spine (Fig. 2E); metatrochanters with large robust spine (Fig. 2F). Abdomen large, AL 0.74–0.92, AW 1.00–1.02. Sternite IX as in Fig. 2I. Aedeagus length 0.39; with long asymmetric median lobe; apical half of parameres membranous (Figs 2J–L).

Female. Similar to male in general; BL 2.60–2.75, HL 0.56–0.58, HW 0.53–0.57, PL 0.56–0.58, PW 0.50–0.52, EL 0.67–0.70, EW 0.98–1.04, AL 0.81–0.89, AW 1.05–1.09. Eyes each composed of about 30 facets. Antennae simple; protibiae not spinose; metaventral horn-like processes absent.

#### Distribution.

 South China: Hainan, Fujian.

#### Comparative notes.

Very distinctive species, easily separable from all other *Pselaphodes* species by the sinuate pro- and metatibiae, the metatibiae with a blade-shaped ventral tooth near apical 1/3, and the serrate ventral margin for the distance posterior to the tooth. The new species shares with *Pselaphodes bomiensis* Yin et al., 2011 and *Pselaphodes condylus* Yin et al., 2011 a similar placement of the antennal modification, but can be readily separated from both by the pronotal lateral margins being rounded. On the contrary the lateral margins of the pronotum are more or less angularly expanded laterally in *Pselaphodes bomiensis* and *Pselaphodes condylus*.

#### Etymology.

 The species name is dedicated to Mei-Ying Lin for the collection of part of the type series.

### Pselaphodes shii

Yin & Li sp. n.

urn:lsid:zoobank.org:act:CE8869A2-3929-4D1B-9D4D-89407D7D026B

http://species-id.net/wiki/Pselaphodes_shii

Figs 3 4

#### Type locality.

 China, Hainan Province, Jianfengling Natural Reserve

#### Type material

(2 ♂♂)**.** HOLOTYPE: ♂, labeled ‘**CHINA:** Hainan Prov. / Ledong County / Jianfengling N. R. / Yulingu, river bank / 18.74686°N, 108.92988°E / ca. 635, 20.iii.2007 / H.L. Shi, F. Yuan leg. // [red] HOLOTYPE / *Pselaphodes shii* Yin & Li / det. 2012, SNUC’. PARATYPE: 1 ♂, labeled ‘**CHINA:** Hainan Prov / Yinggeling, Nankai / Daoyin–Mohao / ca. 335 m, 15.iv.2010 / 19.01021°N, 109.36910°E / Mei-Ying Lin leg. // [yellow] PARATYPE / *Pselaphodes shii* Yin & Li / det. 2012, SNUC’.

#### Diagnosis.

Reddish brown; medium-sized; postocular margins rounded; antennomeres IX strongly modified; pronotum angularly expanded laterally; with robust and long metaventral processes (in lateral view); aedeagus with asymmetric median lobe and long parameres.

#### Description.

**Figure 3.**
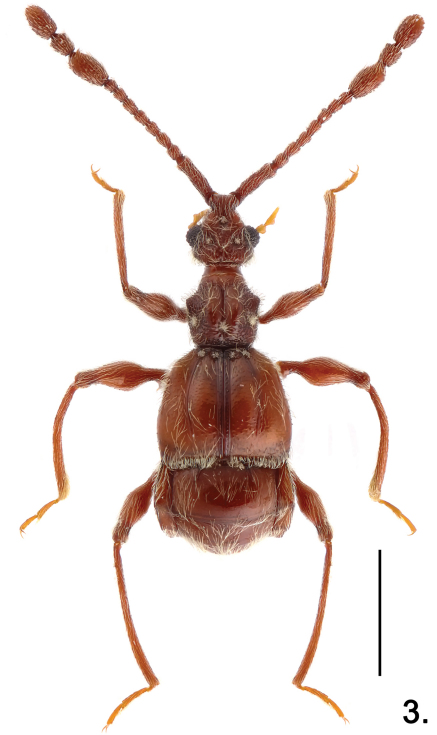
Habitus of *Pselaphodes shii*. Scale: 1.0 mm.

**Figure 4.**
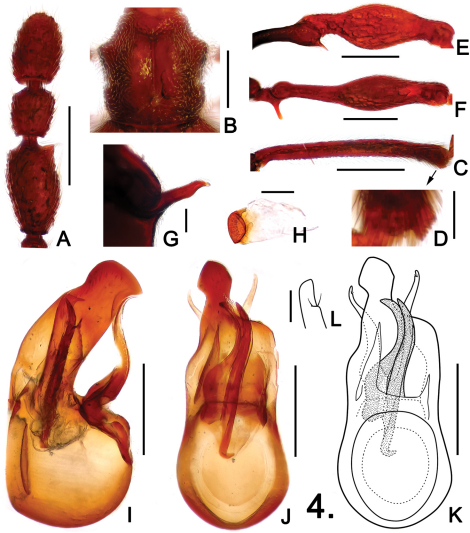
Details of male *Pselaphodes shii*. **A** left antennal club **B** pronotum **C** protibia **D** apex of protibia, enlarged **E** mesotrochanter and mesofemur **F** metatrochanter and metafemur **G** metaventral process, in lateral view **H** sternite IX **I** aedeagus, in lateral view **J–K** same, in dorsal view **L** right paramere, enlarged. Scales: A, B, C, E, F = 0.3 mm, D = 0.05 mm, G, H = 0.1 mm, L = 0.025 mm, I, J, K = 0.2 mm.

 Male (Fig. 3). Length 2.85–2.95. Head as long as wide, HL 0.56–0.60, HW 0.58–0.59; genae rounded; eyes large, each composed of about 40 facets. Antennal clubs as in Fig. 4A. Pronotum (Fig. 4B) almost as long as wide, PL 0.55–0.56, PW 0.57–0.58, with angulate anterolateral margins. Elytra wider than long, EL 0.81–0.84, EW 1.07–1.10. Metaventral horn-like processes long (Fig. 2G). Legs having protibiae with blunt and slightly serrate apical projection (Figs 2C, D); mesotrochanters with distinct ventral spine (Fig. 2E); metatrochanters with long, apically blunt spine (Fig. 2F). Abdomen large, AL 0.93–0.95, AW 1.08–1.12. Sternite IX as in Fig. 2H. Aedeagus length 0.59; with broad asymmetric median lobe and elongate parameres (Figs 4I–K).

Female. Unknown.

#### Distribution.

 South China: Hainan.

#### Comparative notes.

 This species is placed close to *Pselaphodes bomiensis*, *Pselaphodes condylus* and *Pselaphodes jizushanus* Yin et al., 2011 by sharing a similar placement of the antennal modification and projected anterolateral margins of the pronotum. It can be readily separated from *Pselaphodes bomiensis* and *Pselaphodes condylus* by the much smaller size and different form of the antennomeres IX, from *Pselaphodes jizushanus* by the metaventral processes being slightly curved posteriorly, the metatrochanters being protuberant ventrally and the metatibiae being simple. *Pselaphodes jizushanus* has the metaventral processes being curved anteriorly, the metatrochanters are simple, and the metatibiae are bluntly projected at apical 1/5.

#### Etymology.

 The species is named after Hong-Liang Shi for the collection of the holotype.

#### Identification key

The latest key to the Chinese *Pselaphodes* (Yin et al. 2012: 36) is modified as the following to include the two new species described in the present paper.

**Table:** 

11	Metatibiae modified in apical half (Fig. 2G; Yin et al. 2010: 11, Fig. 59; 12, Fig. 70; Yin et al. 2011: 467, Fig. 8)	12
–	Metatibiae simple	14
12	Protibiae with median or apical projection	13a
–	Protibiae lacking projection. (Yunnan)	*Pselaphodes jizushanus* Yin, Li & Zhao, 2011
13a	Protibiae with projection near middle (Yin et al. 2010: 6, Fig. 11). (Anhui, Yunnan, Hainan)	*Pselaphodes aculeus* Yin, Li & Zhao, 2010
-	Protibiae with projection at apex	13
13	Body black (Yin et al. 2010: 4, Fig. 3); antennomeres X slightly transverse (Yin et al. 2010: 13, Fig. 93). (Qinghai)	*Pselaphodes torus* Yin, Li & Zhao, 2010
–	Body reddish brown (Fig. 1); antennomeres X much longer than wide (Fig. 2-A). (Hainan)	*Pselaphodes linae* Yin & Li, sp. n.
14	Metacoxae protuberant	15
–	Metacoxae simple	16
15	Elytra with two pairs of discal humps (Yin et al. 2011: 466, Fig. 6). (Yunnan)	*Pselaphodes gongshanensis* Yin, Li & Zhao, 2011
–	Elytra lacking discal humps (Yin et al. 2012: 32, Fig. 2). (Guangxi)	*Pselaphodes hui* Yin & Li, 2012
16	Mesofemora simple (Yin et al. 2011: 472, Fig. 68; Yin et al. 2010: 12, Fig. 76)	17
–	Mesofemora with tiny admesal spine (Yin et al. 2010: 11, Fig. 61). (Henan)	*Pselaphodes cornutus* Yin, Li & Zhao, 2010
17	Protrochanters simple (Yin et al. 2010: 12, Fig. 70)	17a
–	Protrochanters with distinct ventral spine (Yin et al. 2011: 471, Fig. 51). (Guizhou, Guangxi)	*Pselaphodes condylus* Yin, Li & Zhao, 2010
17a	Large-sized (3.4 mm); antennae and legs conspicuously elongate (Yin et al. 2010: 6, Fig. 12); meso- and metatrochanters simple (Yin et al. 2010: 12, Fig. 76–77). (Yunnan)	*Pselaphodes subtilissimus* Yin, Li & Zhao, 2010
–	Medium-sized (less than 3 mm); antennae and legs normally elongate (Fig. 3); meso- and metatrochanters spinose (Figs 4E, F). (Hainan)	*Pselaphodes shii* Yin & Li, sp. n.

### Taiwanophodes minor

Hlaváč, 2002

http://species-id.net/wiki/Taiwanophodes_minor

Figs 5 6

#### Type locality.

 China, Taiwan, Nantou.

#### Material examined

 (2 ♂♂, 3 ♀♀)**.** 2 ♂♂, labeled ‘**CHINA:** Hainan Prov. / Yinggeling, Nankai / Daoyin–Mohao / ca. 335 m, 15.iv.2010 / Mei-Ying Lin leg.’; 3 ♀♀, labeled ‘**CHINA:** Hainan Prov. / Wuzhishan Mt. / Guzhandao / ca. 640 m, 30.xi.2009 / 18.86657°N, 109.68285°E / Mei-Ying Lin leg.’.

#### Measurements.

Male (female): BL 2.78–2.87 (2.68–2.74), HL 0.56–0.57 (0.57–0.58), HW 0.53–0.54 (0.52–0.54), PL 0.57–0.58 (0.58–0.60), PW 0.55–0.57 (0.53–0.58), EL 0.82–0.84 (0.67–0.69), EW 1.11–1.15 (1.06–0.10), AL 0.83–0.88 (0.86–0.87), AW 1.13–1.16 (1.16–1.22), length of aedeagus 0.50.

#### Diagnosis.

**Figure 5.**
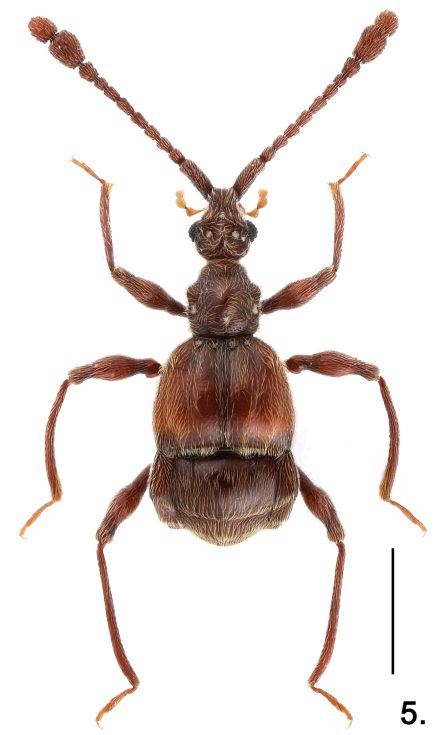
Habitus of *Taiwanophodes minor*. Scale: 1.0 mm.

**Figure 6.**
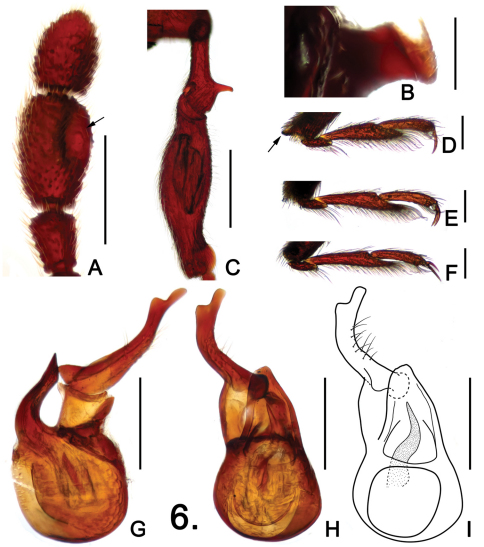
Details of *Taiwanophodes minor*. **A** right antennal club **B** metaventral process, in lateral view **C** protrochanter and profemur **D** protasus **E** mesotarsus **F** metatarsus **G** aedeagus, in lateral view **H–I** aedeagus, in dorsal view. Scales: A, C, G, H, I = 0.3 mm, B, D, E, F = 0.1 mm.

 Male. Reddish brown; medium-sized (Fig. 5); postocular margins rounded; each eye composed of about 40 facets; antennomeres X modified, ventrally concave at apical half (Fig. 6A); pronotum with angulate anterolateral margins; robust and short metaventral processes with hook-like apices (in lateral view) (Fig. 6B); protrochanters spinose ventrally; protibiae with small apical spur (Fig. 6D); tarsomeres II extending to mid-length of tarsomeres III (Figs 6D–F); aedeagus asymmetric, median lobe elongate, parameres largely reduced (Figs 6G–I).

Female. Similar to male in general; each eye composed of about 30 facets; lacking antennal modification; lacking metaventral process; apices of protibiae simple.

#### Distribution.

 South China: Taiwan, Hainan (**New Province Record**).

#### Comparative notes.

 Readily distinguished from the only other congener *Taiwanophodes magnus* Bekchiev, 2010 by the smaller size, antennomeres X being less modified, pronotum being angularly expanded anterolaterally, profemora and protibiae being simple, different conformation of the metaventral processes, and aedeagus with more elongate median lobe and reduced parameres.

#### Comments.

*Taiwanophodes minor* was originally described from central Taiwan (Hlaváč, 2002). It is somewhat surprising that this species also occurs in the Hainan Island, suggesting geographic affinities between the two islands.

## Supplementary Material

XML Treatment for Pselaphodes linae

XML Treatment for Pselaphodes shii

XML Treatment for Taiwanophodes minor

## References

[B1] Bekchiev R (2010) Description of the second species of the genus *Taiwanophodes* Hlaváč, 2002 (Coleoptera: Staphylinidae: Pselaphinae) from Vietnam. Russian Entomological Journal 19 (3): 183-185

[B2] Chandler DS (2001) Biology, morphology, and systematics of the ant-like litter beetles of Australia (Coleoptera: Staphylinidae: Pselaphinae). Memoirs on Entomology International 15: 1-560

[B3] Hlaváč P (2002) A taxonomic revision of the Tyrini of the Oriental region. II. – Systematic study on the genus *Pselaphodes* and its allied genera (Coleoptera: Staphylinidae: Pselaphinae). Annales de la Société Entomologique de France 38 (3): 283-297

[B4] Yin ZW Li LZ Zhao MJ (2010) Taxonomical study on the genus *Pselaphodes* Westwood (Coleoptera: Staphylinidae: Pselaphinae) from China. Part I. Zootaxa 2512: 1-25

[B5] Yin ZW Li LZ Zhao MJ (2011) Taxonomic study on the genus *Pselaphodes* Westwood (Coleoptera, Staphylinidae, Pselaphinae) from China. Part II. Annales Zoologici 61 (3): 463-481 doi: 10.3161/000345411X603337

[B6] Yin ZW Li LZ Zhao MJ (2012) Taxonomic study on the genus *Pselaphodes* Westwood (Coleoptera, Staphylinidae, Pselaphinae) from China. Part III. Zootaxa 3189: 29-38

